# *Potato virus Y* HC-Pro Reduces the ATPase Activity of NtMinD, Which Results in Enlarged Chloroplasts in HC-Pro Transgenic Tobacco

**DOI:** 10.1371/journal.pone.0136210

**Published:** 2015-08-26

**Authors:** Yayi Tu, Zhenqian Zhang, Daofeng Li, Heng Li, Jiangli Dong, Tao Wang

**Affiliations:** State Key Laboratory of Agrobiotechnology, College of Biological Sciences, China Agricultural University, Beijing, P.R. China; Agriculture and Agri-Food Canada, CANADA

## Abstract

*Potato virus Y* (PVY) is an important plant virus and causes great losses every year. Viral infection often leads to abnormal chloroplasts. The first step of chloroplast division is the formation of FtsZ ring (Z-ring), and the placement of Z-ring is coordinated by the Min system in both bacteria and plants. In our lab, the helper-component proteinase (HC-Pro) of PVY was previously found to interact with the chloroplast division protein NtMinD through a yeast two-hybrid screening assay and a bimolecular fluorescence complementation (BiFC) assay *in vivo*. Here, we further investigated the biological significance of the NtMinD/HC-Pro interaction. We purified the NtMinD and HC-Pro proteins using a prokaryotic protein purification system and tested the effect of HC-Pro on the ATPase activity of NtMinD *in vitro*. We found that the ATPase activity of NtMinD was reduced in the presence of HC-Pro. In addition, another important chloroplast division related protein, NtMinE, was cloned from the cDNA of *Nicotiana tabacum*. And the NtMinD/NtMinE interaction site was mapped to the C-terminus of NtMinD, which overlaps the NtMinD/HC-Pro interaction site. Yeast three-hybrid assay demonstrated that HC-Pro competes with NtMinE for binding to NtMinD. HC-Pro was previously reported to accumulate in the chloroplasts of PVY-infected tobacco and we confirmed this result in our present work. The NtMinD/NtMinE interaction is very important in the regulation of chloroplast division. To demonstrate the influence of HC-Pro on chloroplast division, we generated HC-Pro transgenic tobacco with a transit peptide to retarget HC-Pro to the chloroplasts. The HC-Pro transgenic plants showed enlarged chloroplasts. Our present study demonstrated that the interaction between HC-Pro and NtMinD interfered with the function of NtMinD in chloroplast division, which results in enlarged chloroplasts in HC-Pro transgenic tobacco. The HC-Pro/NtMinD interaction may cause the formation of abnormal chloroplasts in PVY-infected plants.

## Introduction

Chloroplasts are believed to have prokaryotic ancestors engulfed by a heterotrophic eukaryotic host cell [1]. They proliferate from pre-existing chloroplasts by binary fission [2]. Bacterial cell division is mediated by the *min* operon [3]. Homologues of components of the bacterial cell division system have been found in chloroplasts [4–7]. Chloroplast division in plants is much more complicated and is mediated by several proteins, both of prokaryotic origin and from the eukaryotic host [8–10]. The first step of chloroplast division is believed to be the formation of Z-ring structure [11]. The Min system ensures proper localization of the Z-ring at the midpoint of chloroplasts [12]. MinD and MinE are two important members of the Min system in chloroplasts. Overexpression of MinD [13] or decreased levels of MinE [14] inhibit chloroplast division. In contrast, reduced levels of MinD [5] or elevated levels of MinE [15] result in multiple chloroplast division sites. AtMinD is a calcium-dependent ATPase and its activity is stimulated by AtMinE *in vivo* [16]. The ATPase activity is critical to release the AtMinD-mediated inhibition of Z-ring formation. AtMinE is a homologue of the topological specificity factor in bacteria [6] that stimulates the ATPase activity of AtMinD at the mid-point of chloroplasts to ensure symmetric division.

*Potato virus Y* (PVY) is a single-stranded, positive-sense RNA virus of the genus *Potyvirus* and enters the top 10 plant virus list for the journal *Molecular Plant Pathology* [17]. PVY infection causes various symptoms, including chlorosis and necrosis associated with changes in chloroplast structure and function [18–22]. Abnormal chloroplasts have been found in virus-infected plants [23, 24] based on their ultrastructure, indicating abnormal formation of chloroplasts in virus-infected plants.

The helper component proteinase (HC-Pro) is one of the 11 mature proteins encoded by PVY [25, 26]. HC-Pro is a multifunctional protein with several suggested roles in the viral infection cycle [27], including involvement in aphid transmission [28, 29], viral cell-to-cell and long-distance movement [30–32], polyprotein processing [33] and suppression of post-transcriptional gene silencing (PTGS) in plants [34–36]. Various host factors have been found to interact with HC-Pro, including a calmodulin-related protein [37]; two novel RING finger proteins, HIP1 and HIP2 [38]; three 20S proteasome subunits, PAA, PBB and PBE [39]; and a calreticulin found in papaya [40]. HC-Pro is also involved in the modulation of host proteosomal catalytic activity [41]. HC-Pro from the genus *Potyvirus* including *Potato virus A* (PVA), *Potato virus Y* (PVY) and *Tobacco etch virus* (TEV) was shown to interact with the translation initiation factor eIF(iso)4E and eIF4E [42]. HC-Pro of *Potato virus A* (PVA) has also been shown to interact with the microtubule-associated host protein HIP2 [43], and mutations in a highly variable region of PVA HC-Pro affect this interaction [44]. Furthermore, HC-Pro interacts with host factors to block RNA silencing [45]. Meanwhile, a calmodulin-like protein in tobacco binds to HC-Pro and direct the degradation of the viral RNA silencing suppressor (RSS) to enhance the host antiviral RNAi system [46].

PVY HC-Pro was reported to be present in the leaf chloroplasts of virus-infected plants [47] and we confirmed this result in our study. The interaction between PVY HC-Pro and the chloroplast division protein NtMinD was verified by our lab in 2007 [48] through a yeast two hybrid screening assay and a bimolecular fluorescence complementation (BiFC) assay *in vivo*. Here, in our present work, we further investigated the biological significance of the NtMinD/HC-Pro interaction. We demonstrated that the ATPase activity of NtMinD was reduced by HC-Pro and HC-Pro competed with NtMinE for binding to NtMinD. HC-Pro transgenic tobacco showed enlarged chloroplasts due to the interaction between HC-Pro and NtMinD. Our present study demonstrated that the interaction between HC-Pro and NtMinD interfered with the function of NtMinD in chloroplast division, which results in enlarged chloroplasts in HC-Pro transgenic tobacco.

## Materials and Methods

### Plant materials and growth conditions

Wild-type *Nicotiana tabacum* tobacco was inoculated with PVY virus through mechanical inoculation. Gloved fingers were wetted with sap from the PVY-infected tobacco, and inoculation was carried out by gently rubbing the bottom leaves with tripolite. PVY-infected plants at 12 days post-inoculation (dpi), together with the wild-type plants, were employed for the detection of the accumulation of HC-Pro in the chloroplasts.

Wild-type and HC-Pro transgenic *N*. *tabacum* plants were grown under standard greenhouse conditions. *N*. *tabacum* was transformed using *Agrobacterium tumefaciens* strain EHA105 via the leaf disc method. A vector containing the leader peptide from the ribulose bisphosphate carboxylase small subunit was constructed, and HC-Pro was cloned into this vector to enable its entry into chloroplasts. The positive transformants of the T_1_ generation were used for chloroplast imaging.

### Protein expression and purification

The HC-Pro gene was cloned from the cDNA of PVY-infected tobacco plants and introduced into the pMAL-c2x vector using the *EcoRI/PstI* restriction sites. NtMinD was cloned into the same vector using the *EcoRI/SalI* restriction sites. The vectors were transformed into *E*.*coli* TB1 cells respectively. IPTG was added at a final concentration of 0.3 mM to induce the production of HC-Pro and NtMinD proteins. Protein induction was performed at 16°C for 14 hours. The culture was collected and ultrasonicated. The lysate was centrifuged at 4°C, 19500 rpm for 30 minutes. The supernatant was loaded onto a column filled with amylose resin to purify the MBP-tagged proteins. The purified proteins were used for *in vitro* biochemical assays.

### Detection of the ATPase activity of NtMinD

The measurement of NtMinD ATPase activity was performed according to Cassie Aldridge and Simon Geir Møller [16] with a few modifications. To test the effect of HC-Pro on the ATPase activity of NtMinD, the two proteins were incubated together for two hours at 4°C to ensure their interaction with each other. After the incubation time, a reaction buffer containing 100 mM Tris-Cl (pH 7.4), 50 mM NaCl, 0.1 mM EDTA, 1.5 mM dithiothreitol, 10% glycerol, 5 mM CaCl_2_ and 10 μM ATP was added. The catalytic reaction was performed at 35°C for 1 hour and terminated with 1 M formic acid. The reaction mixture was transferred to a white COSTAR 96-well plate. The amount of ATP remaining in the reaction mixture was measured using an Adenosine 5’-triphosphate (ATP) Bioluminescent Assay Kit (Sigma Aldrich) according to the manufacturer’s instructions. The strength of luminescent in each well was detected using a Veritas Micorplate Luminometer (Turner BioSystems). Four sets of reactions were conducted and four technical replicates were performed for each sample at the same time on the 96-well plate. Water was used as a negative control, and the plate was pre-read to exclude the background. Data was processed to reflect the ATP hydrolyzed by NtMinD.

### Yeast assays to examine the relationship of NtMinD, NtMinE and HC-Pro

Based on a sequence alignment, the *NtMinE* gene sequence was predicted and cloned from the cDNA of *Nicotiana tabacum*. To determine the interaction site of NtMinD and NtMinE, a yeast two-hybrid assay was employed. Two deletion mutants, NtMinD1 (residues 1–270) and NtMinD2 (residues 271–332), were designed for NtMinD. The coding sequences of the two mutants were cloned into the pGBKT7 vector via the *EcoRI/SalI* site to form the pGBKT7-MinD1 and pGBKT7-MinD2 constructs, respectively. NtMinE was cloned into the pGADT7 vector via the *EcoRI/SacI* site to form the pGADT7-NtMinE plasmid. The pGBKT7-MinD1 and pGBKT7-MinD2 plasmids were separately co-transformed with pGADT7-NtMinE vector into the *Saccharomyces cerevisiae* AH109 cells. Appropriate negative controls were produced in parallel. The interactions were detected on the SD/-Ade/-His/-Leu/-Trp plates. The clones were stained with X-Gal to verify the interaction.

To examine the influence of HC-Pro on the NtMinD/NtMinE interaction, a yeast three-hybrid assay was conducted according to Shan *et al*. with some modifications [49]. *NtMinD* and *HC-Pro* were inserted into the pBridge vector (Clontech) to form the pBridge-NtMinD-HC-Pro construct. The expression of HC-Pro was controlled by the level of methionine (Met). The pBridge-NtMinD-HC-Pro vector was co-transformed with pGADT7-NtMinE vector into the *Saccharomyces cerevisiae* AH109 cells. SD/-Leu/-Trp/-His plates with different concentrations of Met was used to detect the influence of HC-Pro on the NtMinD/NtMinE interaction. Specific β-galactosidase activities were detected with the yeast β-galactosidase assay kit (Thermo Scientific) according to the manufacture’s instructions.

### Chloroplast isolation and protein detection

The chloroplasts of wild-type, PVY-inoculated and HC-Pro transgenic plants were isolated using a chloroplast isolation kit (Sigma-Aldrich) according to the manufacturer’s instructions. Intact chloroplasts were isolated, resuspended in lysis buffer A (50 mM Tris-Cl, pH 8.0; 150 mM NaCl; 0.5% Triton; 1× Protease Inhibitor Cocktail; and 1 mM PMSF) and placed on ice for 10 minutes. Then, the lysed chloroplasts were diluted with buffer B (50 mM Tris-Cl, pH 8.0; 150 mM NaCl; 1× Protease Inhibitor Cocktail; and 1 mM PMSF) and centrifuged at 4°C, 5000 × g for 10 min to separate the membranes and the supernatant. The chloroplast proteins in the supernatant were detected with an antibody raised against HC-Pro through standard western blot procedures.

### Visualization of chloroplasts using DIC imaging

Leaves from the wild-type and HC-Pro transgenic plants were fixed in 3.5% glutaraldehyde in the dark for one hour. The leaves were washed with distilled water three times and incubated in 0.1 M EDTA (pH 9.0) at 55°C for 2 to 3 hours. Samples were viewed using an Olympus BX51 microscope. Measurements were taken using the DP2-BSW (Olympus) software.

## Results

### PVY HC-Pro reduced the ATPase activity of NtMinD

**Fig 1:**
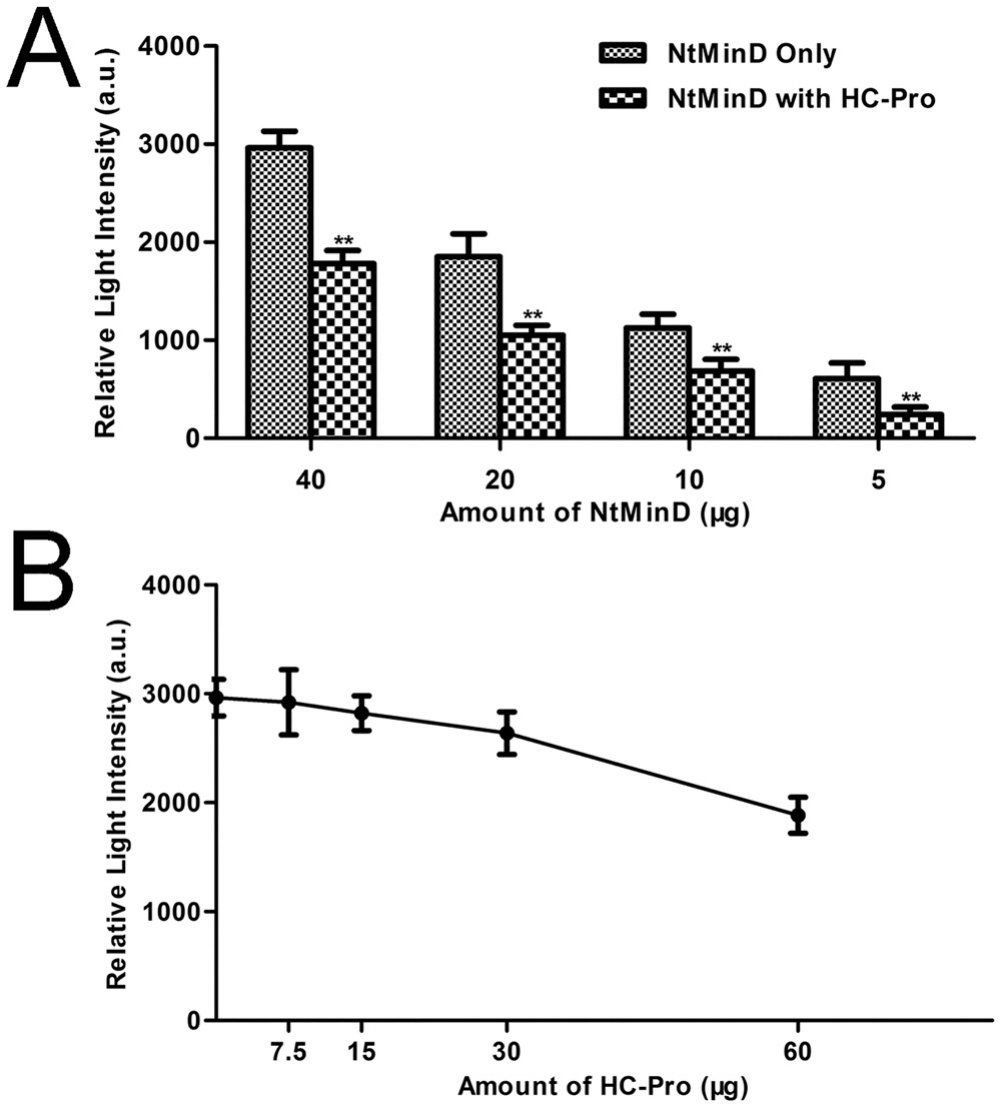
The ATPase activity of NtMinD was reduced by HC-Pro. (A) The same amount of purified HC-Pro (60 μg) was added to various amounts of purified NtMinD (indicated on the X-axis: 40 μg, 20 μg, 10 μg and 5 μg). The relative light intensity indicated on the Y-axis represents the amount of ATP hydrolyzed by NtMinD. NtMinD ATPase activity was significantly reduced by HC-Pro at each amount of NtMinD protein (Student’s *t*-test, **P < 0.01). (B) Various amounts of purified HC-Pro (indicated on the X-axis: 60 μg, 30 μg, 15 μg and 7.5 μg) were added to the same amount of purified NtMinD (40 μg). The relative light intensity indicated on the Y-axis represents the amount of ATP hydrolyzed by NtMinD. The results showed a HC-Pro-mediated dose-dependent reduction of NtMinD ATPase activity.

We previously demonstrated that PVY HC-Pro interacts with NtMinD using yeast two-hybrid and bimolecular fluorescence complementation assays [48]. NtMinD is an ATPase whose enzymatic activity is critical for its role in mediating Z-ring placement *in vivo*. To test whether PVY HC-Pro can influence the enzymatic activity of NtMinD, we purified HC-Pro and NtMinD proteins using the pMAL protein fusion and purification system (New England BioLabs). Purified HC-Pro and NtMinD were combined and incubated for two hours at 4°C, and then the ATPase activity of NtMinD was measured. When the same amount of HC-Pro was added to various amounts of NtMinD, the ATPase activity of NtMinD was reduced in each case (Fig 1A). On the other hand, when various amounts of HC-Pro were added to the same amount of NtMinD protein, a dose-dependent effect was observed: more HC-Pro led to more reduction of the ATPase activity of NtMinD (Fig 1B). These results demonstrate that the ATPase activity of NtMinD is reduced by HC-Pro *in vitro*. The experiment was designed to exclude any background that could interfere with the result of the ATPase activity assay. The reduced ATPase activity of NtMinD was due to HC-Pro, rather than other possible factors.

### Isolation of the topological specificity factor *MinE* gene in *Nicotiana tabacum*

**Fig 2:**
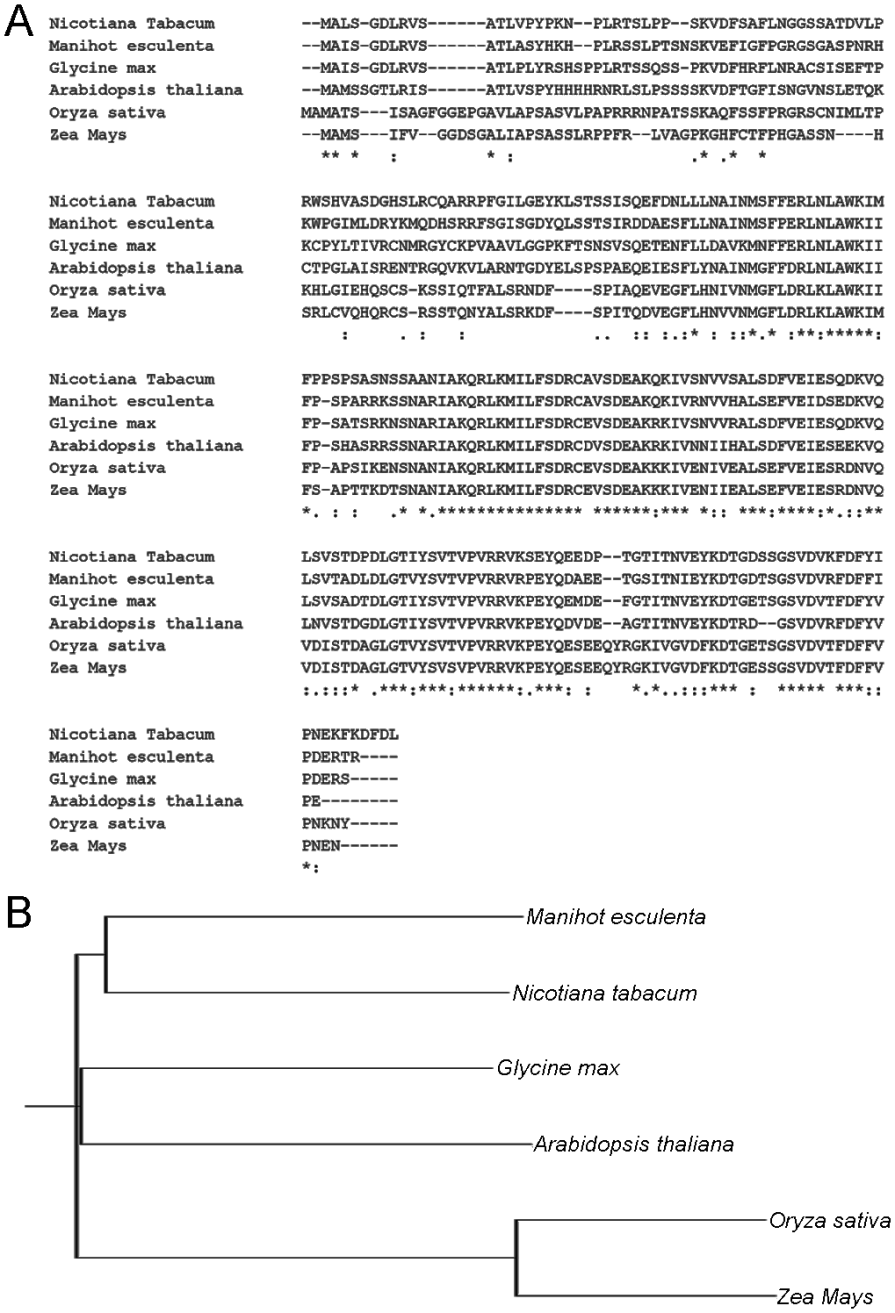
Isolation of *NtMinE* gene in *Nicotiana tabacum*. (A) Sequence alignment of MinE proteins from *Nicotiana tabacum* (accession number KM656074), *Manihot esculenta* (AFC37489.1), *Glycine max* (AAZ23775.1), *Arabidopsis thaliana* (AAG60109.1), *Oryza sativa* (AAT11260.1), and *Zea Mays* (BAD05165.1). (B) Phylogenetic tree analysis among *Nicotiana tabacum*, *Manihot esculenta*, *Glycine max*, *Arabidopsis thaliana*, *Oryza sativa* and *Zea Mays*.

The topological specificity factor MinE is another important component of the chloroplast Min system, which stimulates the ATPase activity of MinD *in vivo* to regulate the Z-ring placement [16]. The full-length cDNA of *NtMinE* was cloned via reverse-transcriptase polymerase chain reaction (RT-PCR). Based on a sequence alignment with *MinE* from *Arabidopsis*, *Manihot esculenta*, *Glycine max*, *Oryza sativa* and *Zea Mays*, we concluded that the cloned gene encodes the tobacco MinE protein (Fig 2A). The sequence of this putative chloroplast division-related gene, named *NtMinE*, has been submitted to GenBank (accession number KM656074). Analysis of the phylogenetic tree demonstrated that NtMinE has the highest similarity with Manihot esculenta among the six different MinE proteins (Fig 2B).

### HC-Pro competes with NtMinE for binding to NtMinD

**Fig 3:**
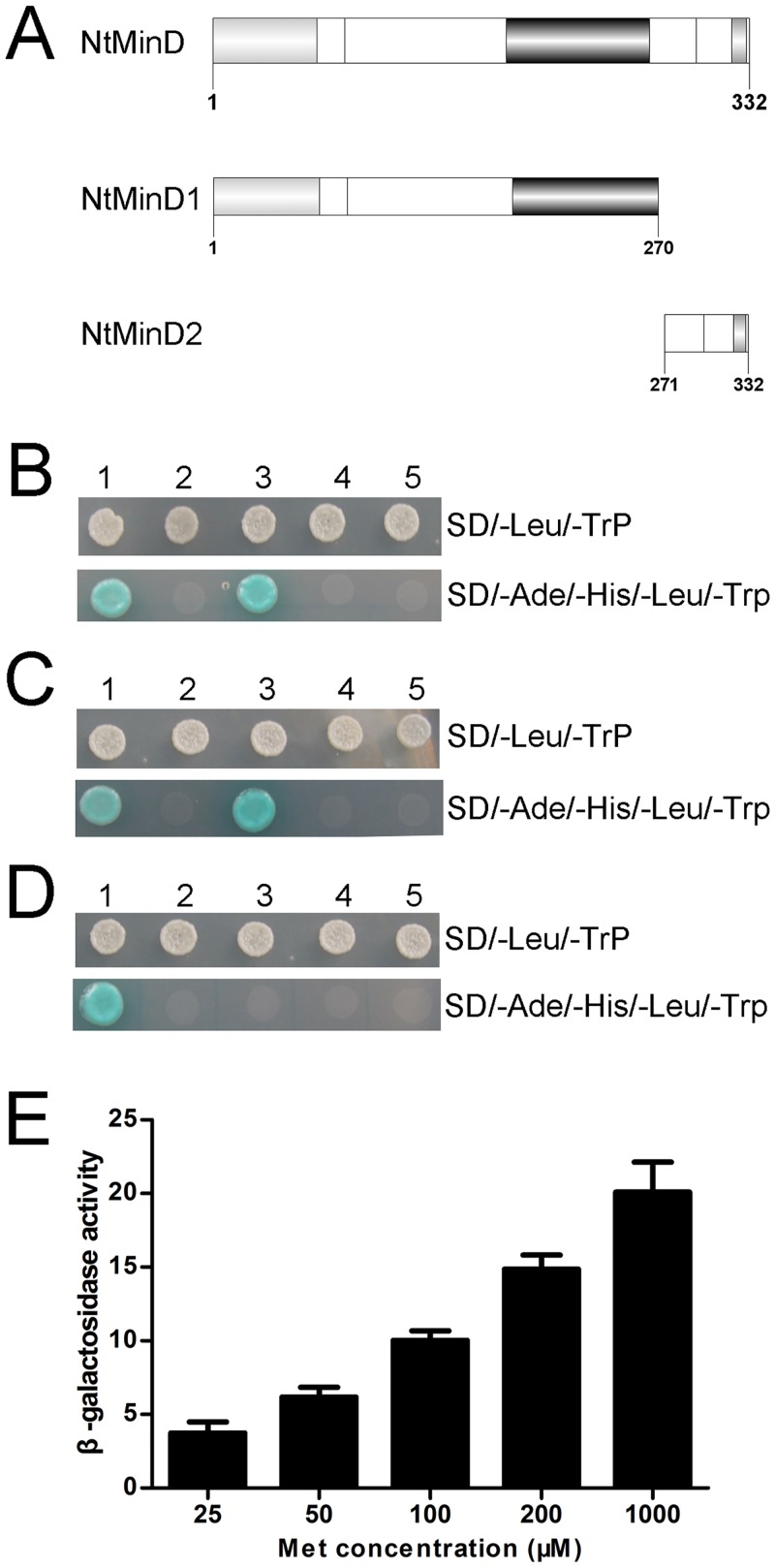
HC-Pro interfered with the NtMinD/NtMinE interaction. (A) Schematic overview of the NtMinD domains and deletion mutants. The NtMinD1 mutant contains amino acids 1–270, and the NtMinD2 mutant contains amino acids 271–332. The mutants were designed to determine the NtMinD domains required for interaction with NtMinE. (B) Interaction of NtMinE with the full-length of NtMinD. 1, pGADT7-RecT /pGBKT7-53 (positive control); 2, pGADT7/pGBKT7 (negative control). 3, pGADT7-NtMinE/pGBKT7-NtMinD; 4, pGADT7-NtMinE/pGBKT7; 5, pGADT7 /pGBKT7-NtMinD. The results indicated that NtMinE interacted with the full-length NtMinD. (C) Interaction of NtMinE with the NtMinD2 mutant. 1, pGADT7-RecT /pGBKT7-53 (positive control); 2, pGADT7/pGBKT7 (negative control). 3, pGADT7-NtMinE/pGBKT7-NtMinD2; 4, pGADT7-NtMinE/pGBKT7; 5, pGADT7 /pGBKT7-NtMinD2. The results indicated that NtMinE interacted with amino acids 271–332 of NtMinD, which overlapped the HC-Pro interaction site. (D) Interaction of NtMinE with the NtMinD1 mutant. 1, pGADT7-RecT /pGBKT7-53 (positive control); 2, pGADT7/pGBKT7 (negative control). 3, pGADT7-NtMinE/pGBKT7-NtMinD1; 4, pGADT7-NtMinE/pGBKT7; 5, pGADT7 /pGBKT7-NtMinD1. The results indicated that NtMinE was unable to interact with amino acids 1–270 of NtMinD. (E) Yeast three-hybrid assay of NtMinD, NtMinE and HC-Pro. The influence of HC-Pro on the NtMinD/NtMinE interaction was indicated by β-galactosidase activity on the Y-axis. Different concentrations of Met were indicated on the X-axis. Higher level of HC-Pro at lower Met concentration led to lower β-galactosidase activity, demonstrating an inhibition of HC-Pro on the NtMinD/NtMinE interaction.

The *in vivo* interaction between MinD and MinE is important for the biological role of MinD in regulating the Z-ring placement. We determined the interaction site of NtMinD and NtMinE using a yeast two-hybrid assay. Two deletion mutants of NtMinD were used: NtMinD1 (residues 1 to 270), which contained a transit sequence and ParA family conserved domain; NtMinD2 (residues 271 to 332), which contained the NtMinD/HC-Pro interaction site (Fig 3A). Full-length NtMinE was sub-cloned into the pGADT7 vector using the *EcoRI/SacI* sites to form the pGADT7-NtMinE plasmid. Full-length NtMinD and the two deletion mutants were sub-cloned individually into the pGBKT7 vector using the *EcoRI/SalI* sites. The pGADT7-NtMinE plasmid was co-transformed into *Saccharomyces cerevisiae* AH109 cells along with pGBKT7-NtMinD, pGBKT7-NtMinD1 or pGBKT7-NtMinD2 respectively. The results indicated that NtMinE interacts with full-length NtMinD (Fig 3B). In addition, the NtMinE/NtMinD2 transformant grew on the SD/-Ade/-His/-Leu/-Trp medium (Fig 3C), but the NtMinE/NtMinD1 transformant did not (Fig 3D). These results showed that NtMinE interacts with the C-terminus of NtMinD, which overlaps the NtMinD/HC-Pro interaction site at amino acids 297–314 of NtMinD [48].

To further investigate the relationship of NtMinD, NtMinE and HC-Pro, we conducted a yeast three-hybrid assay. The expression level of HC-Pro was controlled by the level of Met in the medium; i.e., HC-Pro was repressed in the presence of 1000 μM Met and expressed in the absence of Met. The results showed that the NtMinD/NtMinE interaction was gradually weakened by increasing concentrations of HC-Pro (Fig 3E). The results suggested that in addition to reducing the ATPase activity of NtMinD, HC-Pro competes with NtMinE for binding to NtMinD.

### Chloroplasts in HC-Pro transgenic plants were enlarged

**Fig 4:**
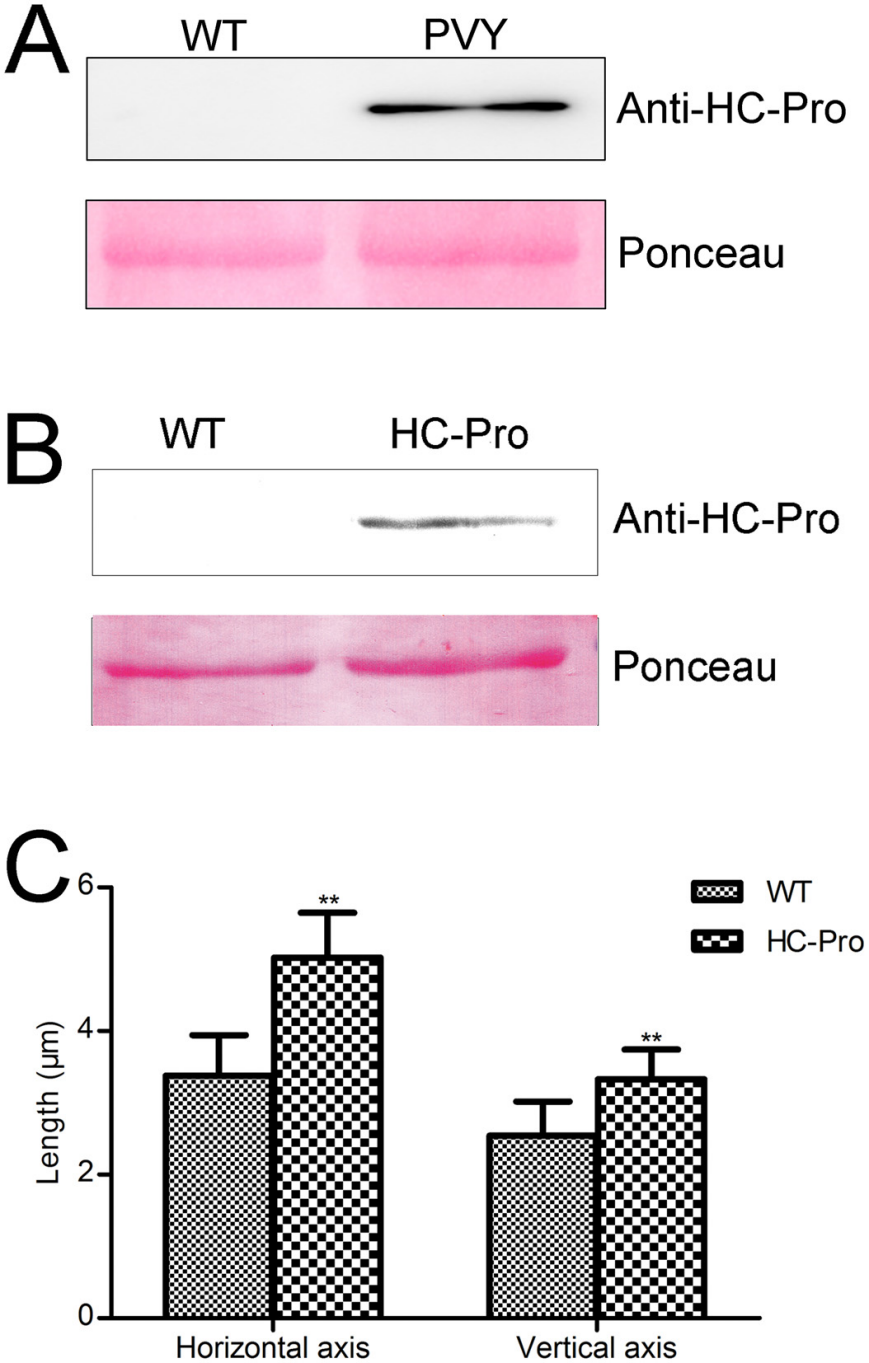
Chloroplasts were enlarged in the HC-Pro transgenic tobacco plants. (A) Western blot of wild-type (WT) and PVY-infected (PVY) tobacco using chloroplast proteins. Intact chloroplasts were isolated and chloroplast proteins were extracted from the intact chloroplasts. Antibody raised against HC-Pro (Anti-HC-Pro) was used to detect HC-Pro accumulation in the chloroplasts. Ponceau staining of the large subunit of Rubisco was used as a loading control. (B) Western blot of wild-type (WT) and HC-Pro transgenic plants (HC-Pro) using chloroplast proteins. Intact chloroplasts were isolated from fresh leaves of wild-type and HC-Pro transgenic plants. Chloroplast proteins were extracted from the intact chloroplasts. The antibody raised against HC-Pro (Anti-HC-Pro) was used to detect HC-Pro accumulation in the chloroplasts. Ponceau staining of the large subunit of Rubisco was used as a loading control. (C) Statistical data of the horizontal and vertical axis of chloroplasts in wild type (WT) and HC-Pro transgenic plants (HC-Pro). Both the horizontal and vertical axis of chloroplasts in the HC-Pro transgenic plants were significantly longer than those of the chloroplasts in wild-type plants (Student’s t-test, **P < 0.01), indicating that the chloroplasts in HC-Pro transgenic plants were enlarged. Over 60 chloroplasts were measured from both the wild-type and HC-Pro transgenic plants.

The accumulation of HC-Pro in the chloroplasts of PVY-infected tobacco was reported by Gunasinghe and Berger in 1991 [47] and we confirmed this result in our study. Intact chloroplasts of wild-type, mock-inoculated and PVY-infected tobacco were isolated and the chloroplast proteins were extracted. Western blot analysis indicated that HC-Pro accumulates in the chloroplasts of PVY-infected tobacco, but not in the wild-type plants (Fig 4A). As an important component of the chloroplast Min system, NtMinD associates with the membrane in its ATP-binding form and inhibits Z-ring formation. When the ATPase activity of this factor is stimulated by NtMinE, NtMinD dissociates from the membrane in its ADP-binding form and releases its inhibition of Z-ring formation. Z-ring assembles at the site with the lowest NtMinD concentration. The above results demonstrated that the ATPase activity of NtMinD was reduced by HC-Pro. And HC-Pro competes with NtMinE for binding to NtMinD. We assume that HC-Pro is responsible for enlarged chloroplasts. To determine the role of HC-Pro in the formation of abnormal chloroplasts, HC-Pro transgenic tobacco plants were generated and their chloroplasts were analyzed. HC-Pro was retargeted to the chloroplasts using the transit peptide of the ribulose bisphosphate carboxylase small subunit (rbcs). The chloroplasts of the transgenic plants were isolated and the chloroplast proteins were extracted for the western blotting analysis. The results showed that HC-Pro was successfully accumulated in the chloroplasts (Fig 4B). Fully expanded leaves of the transgenic plants were used to visualize the chloroplasts. The length and width of the chloroplasts were measured, and statistical data indicated that chloroplasts were enlarged in the transgenic plants (Fig 4C).

## Discussion

PVY is an important plant virus responsible for great losses worldwide. The protein-protein interaction network of the plant-virus pathosystem is very complicated [50]. HC-Pro was found to accumulate in the chloroplasts of PVY-infected tobacco plants [47] and this result was confirmed through western-blot of the chloroplast proteins from PVY-infected plants in our study. The accumulation of PVY HC-Pro in the chloroplasts of infected tobacco plants [47] indicates some other functions of HC-Pro in addition to its multiple functions in the cytoplasm. Plants infected by PVY often show abnormal chloroplasts [23, 24]. Using the yeast two-hybrid system and bimolecular fluorescence complementation assays, the interaction between HC-Pro and NtMinD was previously confirmed in our lab [48]. Here, the biological significance of this interaction was investigated. We demonstrated that HC-Pro reduces the ATPase activity of NtMinD and enlarged chloroplasts are observed in HC-Pro transgenic tobacco.

**Fig 5:**
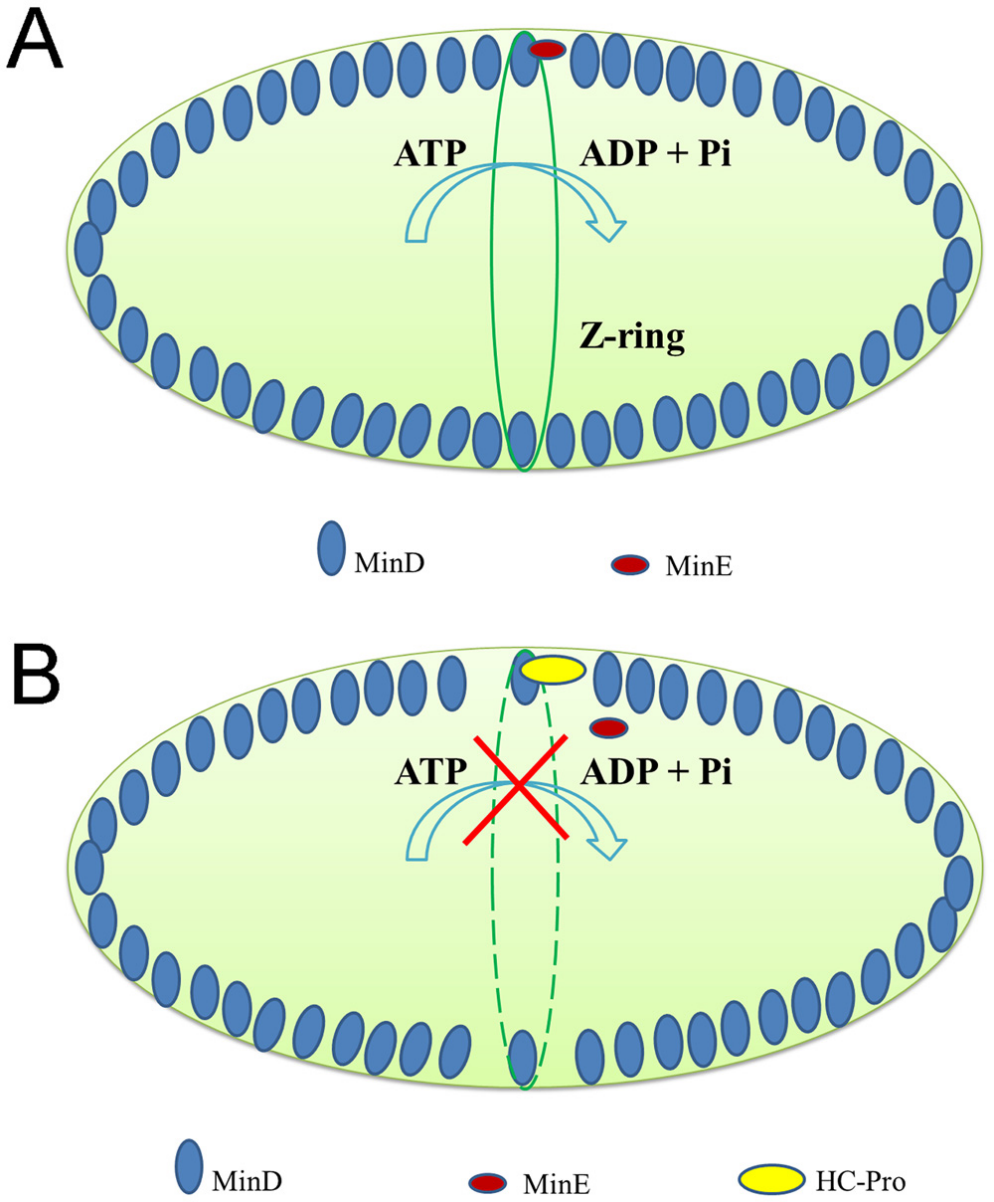
Model of the inhibition of chloroplast division by HC-Pro. (A) In wild-type plants, the ATPase activity of NtMinD is stimulated by NtMinE. The Z-ring is formed at the middle of chloroplasts and normal chloroplast division occurs. (B) In HC-Pro transgenic plants, the ATPase activity of NtMinD is inhibited by HC-Pro. The Z-ring fails to assemble and the chloroplast division is arrested, leading to enlarged chloroplasts.

In wild type plants, MinD binds to the inner envelop membrane (IEM) of chloroplasts and inhibits Z-ring formation [8]. Another important chloroplast division protein found in plants, MinE, shows the same localization [15]. The coordination of these two proteins leads to the symmetric division of chloroplasts [51]. As a topological specificity factor, NtMinE stimulates the ATPase activity of NtMinD *in vivo*, and the energy from ATP hydrolysis is used to release NtMinD from the IEM, causing the lowest NtMinD concentration at the mid-point of chloroplasts. Thus, under normal conditions, the Z-ring assembles at the mid-point of chloroplasts, and symmetric division of chloroplasts occurs (Fig 5A). However, in HC-Pro transgenic plants, the NtMinD/HC-Pro interaction can interfere with the Z-ring assembly. HC-Pro competes with NtMinE for binding to NtMinD *in vivo* and the ATPase activity of NtMinD is reduced by HC-Pro, in which case ATP hydrolysis is inhibited and there will be no energy for NtMinD to release from the IEM. Thus, the assembly of Z-ring is disturbed, and the chloroplasts become enlarged (Fig 5B).

Chloroplasts are main targets for viral attack [52]. Viral infections often cause abnormal chloroplast function and morphology. Due to the influence of HC-Pro on the ATPase activity of NtMinD, the chloroplast division defect in the HC-Pro transgenic plants resembles the effects of overexpression of NtMinD and mutation of NtMinE [13, 51]. However, the phenotype was not as severe; we did not see one large chloroplast in a single cell or extremely large chloroplasts. We believe that the milder phenotype observed here is the result of incomplete inhibition of the NtMinD ATPase activity by HC-Pro. Nevertheless, this inhibition is important in the formation of enlarged chloroplasts. The enlarged size of chloroplast must cause some disturbance in normal chloroplast function. HC-Pro is usually thought to be a suppressor of RNAi in plants and to carry out its functions in the cytoplasm. Here, we find a new function for this protein in the chloroplasts. Our findings here demonstrated the interference of the function of NtMinD by HC-Pro and revealed the reason for abnormal chloroplast formation in PVY-infected plants at the molecular level.
